# Cementless TKA use as an alternative to cemented TKA in high BMI patients: A systematic review and meta‐analysis

**DOI:** 10.1002/jeo2.12067

**Published:** 2024-07-15

**Authors:** Giang Truong Le, Bernard Hendrick van Duren, Kevin Ilo, Reshid Berber, Hosam E. Matar, Benjamin V. Bloch

**Affiliations:** ^1^ Nottingham Elective Orthopaedic Services Nottingham University Hospitals NHS Trust Nottingham UK; ^2^ Leeds Institute of Rheumatic and Musculoskeletal Medicine University of Leeds Leeds UK

**Keywords:** cemented, cementless, high BMI, obese, TKA

## Abstract

**Purpose:**

Obesity is prevalent, with nearly one‐third of the world's population being classified as obese. In patients with high body mass index (BMI)/body mass undergoing total knee arthroplasty (TKA), there is an increase in strain placed on the implant fixation interfaces. As such, component fixation is a potential concern when performing TKA in the obese patient. To address the growing concerns around the longevity of implant fixation, some have advocated cementless over cemented fixation. However, there is no clear consensus on whether a cementless fixation has more favourable outcomes. The aim of this paper was to present a systematic review and meta‐analysis of the existing evidence to establish if cementless TKA has a lower rate of aseptic loosening in high BMI patients when compared to cemented TKA procedures.

**Methods:**

A systematic review was performed, and the following databases Medical Literature Analysis and Retrieval System Online (1946 to date), PubMed (1966 to date) and Excerpta Medica Database (1974 to date) were searched. All studies comparing cementless to cemented TKA in patients with BMI > 30 were considered. Meta‐analysis compared aseptic loosening and all‐cause revision between cemented and uncemented implant use in BMI > 30 patients.

**Results:**

The search returned 91 articles in total; after duplicates were removed, the yield was 44 studies. Of the remaining studies that were assessed, three studies met the inclusion criteria for meta‐analysis. The pooled odds ratio for all‐cause revisions was 0.17 (95%, 0.08–0.36) in favour of uncemented implants (*p* < 0.01). The pooled odds ratio for aseptic loosening was 0.15 (95% confidence interval, 0.02–0.90) in favour of uncemented implants (*p* = 0.04).

**Conclusions:**

Meta‐analysis demonstrated a significant decrease in all‐cause revisions and revisions for aseptic loosening when using uncemented fixation in high BMI patients when compared to the use of cemented implants.

**Level of Evidence:**

The level of evidence is 1 for our systematic review.

**Abbreviations**
AHRQ: agency for healthcare research and quality
BMI: body mass index
EMBASE: Excerpta Medica Database
GRADE: Grading of Recommendations, Assessment, Development and Evaluations
MEDLINE: Medical Literature Analysis and Retrieval System Online
NOS: Newcastle–Ottawa Scale
OCEBM: Oxford Centre for Evidence‐Based Medicine
PICO: Population Intervention Comparison Outcome
PRISMA: Preferred Reporting Items for Systematic Reviews and Meta‐Analyses
PROM: patient‐reported outcome measure
RCT: randomised controlled trial
TKA: total knee arthroplasty
USA: United States of America

## INTRODUCTION

Obesity is defined as a body mass index (BMI) greater than or equal to 30 [29]. It is a major risk factor for developing osteoarthritis of the weight‐bearing joints such as the hips and knees, which is a common indication for primary arthroplasty [24]. The prevalence of obesity has almost tripled worldwide since 1975, and 13% of adults were classified as obese in 2016 [29]. Approximately, a third of the global population is either obese or overweight according to a more recent article [9]. It has been estimated that 51% of the global population will be either overweight or obese by 2035 [29]. In Western societies like England and the United States, the percentage of adults who are obese are 25.9% and 41.9%, respectively [6, 37].

Obesity poses unique challenges during total knee arthroplasty (TKA) surgery due to various anatomical factors and co‐morbidities. Excessive joint loading due to obesity exacerbates joint malalignment and cartilage degeneration [5]. Obesity together with insulin resistance and dyslipidaemia are associated with increased levels of acute phase reactants and cytokines resulting in meta‐inflammation [10]. However, the causal relationship between these factors is yet to be established [10]. Obese patients undergoing TKA are at increased risk of developing complications such as infection and aseptic loosening [20]. However, even with these challenges, high BMI patients achieve similar outcomes to nonobese patients [13], and it has been shown that TKA remains cost‐effective for the obese patient [12].

Patients with a BMI of over 40 have been shown to have a revision rate of 7% within 5 years compared to 2% for nonobese patients [3]. The most common indication for revision is aseptic loosening which accounts for 47% of revision cases [28]. Aseptic tibial loosening is the most common cause of failure in TKA; [32] the risk has been shown to be twice as likely for patients with a BMI of over 35 [18].

In patients with high BMI, increased forces are placed across the implant fixation interfaces [1, 26]. As such, component fixation and the potential for aseptic loosening has been a concern when performing TKA in the obese patient. It has been suggested that cementless implants may reduce the risk of aseptic loosening when compared to conventional cemented implants in high BMI patients [36]. Cementless knee implants are designed with a porous surface which enables bony ingrowth, effectively eliminating an interface [30]. This is thought to provide a more long‐term biologic fixation where there are concerns the cement may have a degradation effect over time causing the implant to loosen and form cement debris which may induce an osteolytic reaction [16].

For obese patients requiring a TKA, there is no clear consensus on whether a cementless fixation has more favourable outcomes. This paper presents a systematic review and meta‐analysis of the existing evidence to establish if cementless TKA has a lower rate of aseptic loosening in high BMI patients when compared to cemented TKA procedures.

## METHODS

This systematic review and meta‐analysis were conducted along Preferred Reporting Items for Systematic Reviews and Meta‐analyses guidelines. A systematic search was performed using the following databases: Medical Literature Analysis and Retrieval System Online (1946 to date), PubMed (1966 to date) and Excerpta Medica Database (1974 to date). ‘Total knee replacement, total knee arthroplasty’, ‘obese, obesity, overweight, BMI, body mass index’, ‘cementless, uncemented’ and ‘aseptic loosening’ were used as keywords in connection with AND or OR.

Inclusion criteria were established following the Population Intervention Comparison Outcomes) approach:

- Population: patients with BMI ≥ 30 who had undergone TKA.
- Intervention: cementless primary TKA.
- Comparison: cemented primary TKA.
- Outcome: aseptic loosening, all causes of revision, complications, functional scores.

Only articles published in English were included. Titles and abstracts were screened for relevance prior to full inspection. The studies were independently assessed by two reviewers; any discrepancies were mutually agreed upon after discussion. Full texts of the manuscripts meeting the inclusion criteria were obtained.

Data were extracted using a standardised data collection form, which was first tested using sample articles and adjusted to optimise efficiency and accuracy prior to final data collection. The following data were recorded: (a) demographics: population studied, age, gender, surgery, side, indication; (b) study characteristics: study design, number of subjects, randomisation, blinding and (c) outcomes: aseptic loosening, all causes of revision, complications, functional scores.

### Analysis

The extracted data were analysed using the statistical software Review Manager version 5.3 (Cochrane). Aseptic loosening and all‐cause revision were compared between cemented and uncemented implant use in obese patients. Results from individual studies were pooled by meta‐analysis using the Mantel–Haenzel method [25]. A random effects model was used as heterogeneity between studies from clinical or methodological diversity was considered likely, and the *I* ^2^ values were greater than 25%.

### Assessment of methodological quality and risk of bias

The Newcastle–Ottawa Scale (NOS) was used to assess the quality of the nonrandomised studies included in our meta‐analysis by analysing the selection of study groups, comparability and the outcomes [40].

The overall quality of the evidence in the meta‐analyses was assessed using the Grading of Recommendations, Assessment, Development and Evaluation (GRADE) system [35]. Recommendations were classified as either high, moderate, low or very low (high = we are very confident that the effect in the study reflects the actual effect, moderate = we are quite confident that the effect in the study is close to the true effect, but it is also possible it is substantially different, low = the true effect may differ significantly from the estimate, very low = the true effect is likely to differ significantly from the estimate). This approach involved grading the evidence based on the criteria below:

1. Study design: randomised trial = high, observational study = low, any other evidence = very low.
2. Study quality (based on NOS scores): high, moderate, low or very low.
3. Inconsistency of results: assessment was based on the *I* ^2^ value (downgraded if the *I* ^2^ was >25% indicating high heterogeneity and there was no plausible explanation).
4. Indirectness.
5. Imprecision of results: downgraded if the 95% confidence interval (CI) did not exclude the baseline.
6. Publication bias (assessed using funnel plot analysis): downgraded if bias was determined.

## RESULTS

### Included studies

**Figure 1:**
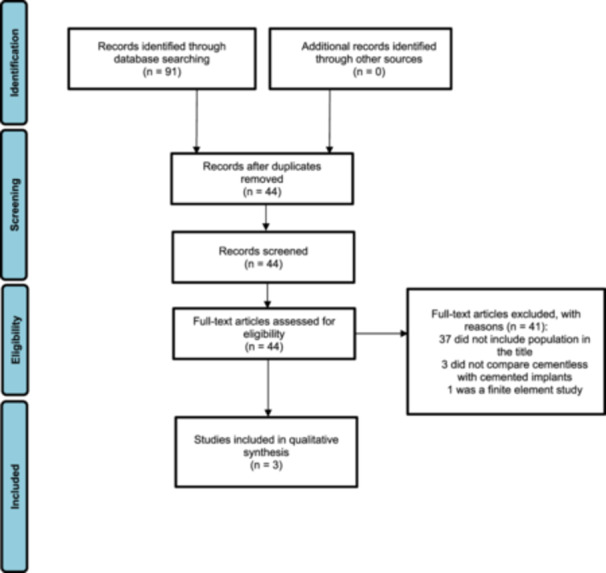
Preferred Reporting Items for Systematic Reviews and Meta‐Analyses diagram.

Our literature search initially identified 91 studies in total; after duplicates were removed, the yield was 44 studies. Of the remaining studies that were assessed, three studies met the eligibility criteria to be included in the meta‐analysis [2, 4, 36] (Figure 1). Thirty‐seven studies were deemed not eligible as they did not include obese patients in their titles. Three studies did not directly compare outcomes from the cementless with the cemented implant cohorts and so were removed [17, 21, 34]. One study was a finite element study which was not relevant to this meta‐analysis [39].

### Study characteristics

There were no relevant randomised control trials (RCTs) identified in the literature. All the included studies were retrospective cohort studies, published between 2016 and 2019. Data of all‐cause revision and revision for aseptic loosening was available for all studies. Patient‐reported outcome measures (PROMs) were only measured in two of the studies but could not be directly compared as different scoring systems were used (Knee Society Score [22], the Lower Extremity Activity Scale [33] and the Forgotten Joint Score‐12 [31]). The scores could not be converted into a common scoring system as the individual data for each component were not available.

### Patient characteristics

A total of 817 patients were included of which 407 had undergone cementless TKA in comparison to 410 who had cemented TKA. Two of the studies included morbidly obese patients with BMI ≥ 40 [2, 36], and the remaining study included obese patients with BMI ≥ 30 [4]. The mean BMI was 42.1 (range 30.1–66), and the mean age was 61.6 years (range 39–80). Two hundred and nineteen of the patients were male and 598 were female. Preoperative diagnoses of diabetes mellitus were recorded in one of the included studies [4]. The follow‐up times from the studies ranged from 2 to 5 years.

### All‐cause revision

**Figure 2:**
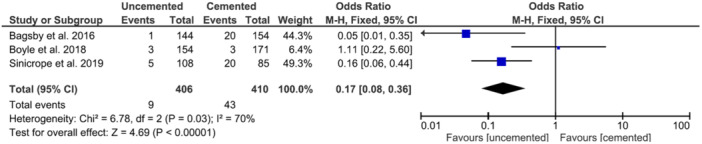
Forest plot of odds ratios for all‐cause revisions. CI, confidence interval.

The pooled odds ratio for all‐cause revisions was 0.17 (95%, 0.08–0.36) in favour of uncemented implants (Figure 2). This finding was significant (*p* < 0.01).

### Revision for aseptic loosening

**Figure 3:**
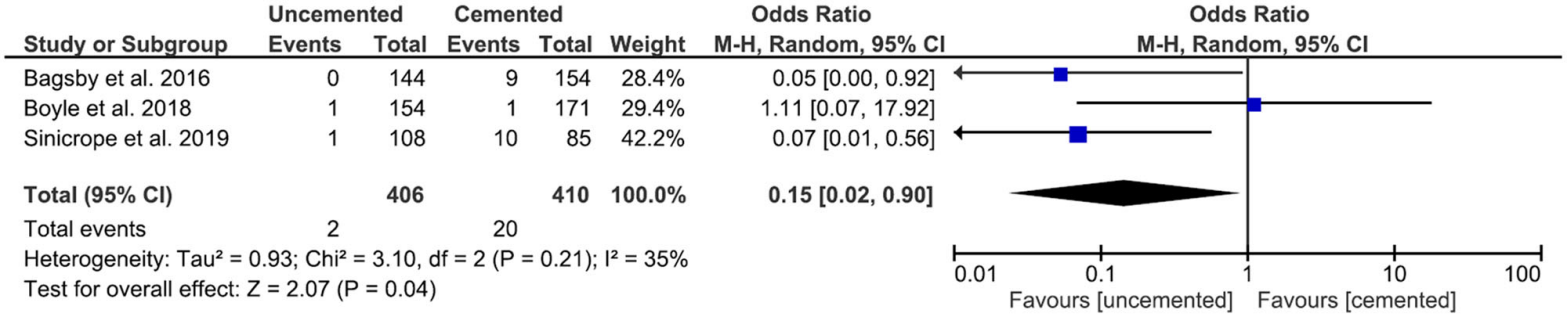
Forest plot of odds ratios for aseptic loosening. CI, confidence interval.

The pooled odds ratio for aseptic loosening was 0.15 (95% CI, 0.02–0.90) in favour of uncemented implants. This finding was significant (*p* = 0.04) (Figure 3).

### Assessment of methodological quality and risk of bias

All the included studies were cohort studies which are graded as level IV evidence on the Oxford Centre for Evidence‐Based Medicine scale [7]. The retrospective nature of the cohort studies meant that the results were susceptible to information bias, and we had less control over the variables that were measured.

Table 1 summarises the assessment of methodological quality using the NOS [40]. All the included studies were rated as poor quality by agency for healthcare research and quality standards as they did not demonstrate evidence of using stratification or multivariate models to adjust for potential confounding factors. GRADE analysis found the quality of evidence to be very low or low for all analyses [35]. Based on this, there is a possibility that the true effect may differ significantly from the estimates presented in Figures 2 and 3.

**Table 1:** Newcastle–Ottawa Scale measurement of methodological quality.

References	Selection (out of 4 stars)	Comparability (out of 2 stars)	Outcome (out of 3 stars)	AHRQ standard
Sinicrope et al. [36]	4	0	3	Poor
Boyle et al. [4]	4	0	3	Poor
Bagsby et al. [2]	4	0	3	Poor

Abbreviation: AHRQ, agency for healthcare research and quality.

## DISCUSSION

Our results showed that there was a significant decreased risk of all‐cause revisions and aseptic loosening in obese patients who received an uncemented implant as opposed to a cemented implant. To our knowledge, this meta‐analysis is the first to compare the use of cementless to cemented implants in obese patients.

The existing literature shows that TKA in obese patients can be more technically demanding and associated with worse outcomes when compared with their nonobese counterparts [3]. Obese patients have a higher risk of developing wound infections and prosthetic joint infections following TKA [20]. There is evidence to suggest this may be due to increased surgery time owing to operative difficulty [14], a reduced immune response [23] and reduced subcutaneous tissue oxygenation [19]. Obese patients undergoing TKA have been shown to have higher revision rates [3]. The purported mechanism is that greater weight loading across the tibial component leads to increased loosening and therefore poorer implant survival due to mechanical failure [18]. Morbidly obese patients were found to have a longer length of stay and higher readmission rates within 1 month after TKA [8]. Nevertheless, at 2 years follow‐up, this cohort was shown to have a large improvement in Oxford Knee Scores and Knee Society Knee Scores [8].

The finite element analysis paper which did not meet the inclusion criteria for meta‐analysis showed that patients with higher BMI caused additional strain and micromotion between the prosthesis and femur; this suggested certain gait activities could pose a risk for prosthesis stability in this population [39]. Cementless implants in knee arthroplasty enable stronger component fixation for obese patients as the porous implant covering can integrate directly with the bone to provide a mechanical interlock [38]. This provides other benefits such as the preservation of bone stock, reduced radiological radiolucent lines associated with aseptic loosening and reduced side effects related to cement debris [11]. A study of the normal cohort concluded that long‐term implant survivorship at 10 and 20 years showed no significant difference between cementless and cemented TKAs [27].

It is worth noting that Boyle et al. [4] showed inconclusive odds ratios in both outcome measures; this was partly due to the limited sample size and number of events recorded compared to the other studies included. A systematic review comparing morbidly obese patients and nonobese patients undergoing TKA showed that revision rates were 7% and 2%, respectively, [3] which supports the conclusions of the other studies which met our inclusion criteria [2, 36].

Our study was limited by the sample size which included a total of 817 patients from both cemented and uncemented cohorts over three studies. Furthermore, BMI over 30 was used in Boyle et al. [4] as opposed to BMI over 40 in the other two studies [2, 36]. Boyle et al. [4] showed there was no statistically significant difference between the rate of all‐cause revision or aseptic loosening between the uncemented and cemented groups. Conversely, the other two studies [2, 36] showed a statistically significant difference in these outcome measures when only morbidly obese patients were included. Mean BMI in the different groups was recorded rather than BMI as a continuous variable; therefore, it was not possible to ascertain a specific BMI measurement beyond which the increased risk of outcome measures became significant.

The mean time to revision was not reported in the included studies which meant we could not comment on whether revision surgeries were required in the short, medium or long term. Sinicrope et al. [36] showed 99.1% survivorship at the 8‐year follow‐up of aseptic loosening following cementless TKA in morbidly obese patients compared to 88.2% in the cemented cohort. Bagsby et al. [2] recorded a mean follow‐up of 6.1 and 3.6 years for the cemented and uncemented groups, respectively. This study recorded nine cases of aseptic loosening in the cemented group compared to none in the uncemented group. Boyle et al. [4] recorded no statistically significant difference in survivorship for aseptic loosening of the tibial component at a mean follow‐up of 5.7 years for both the cemented and uncemented groups with a BMI over 30. Another retrospective study evaluating the outcomes following a posterior‐stabilised, rotating platform cementless TKA in obese and nonobese patients showed no significant difference in complications, revisions or 10‐year survival [15]. The relatively short follow‐ups of the included studies is a limitation of our meta‐analysis.

Bagsby et al. [2] showed that Knee Society Scores [22] were significantly higher in the cementless group which indicates improved PROMs relating to functionality and pain. However, Boyle et al. [4] showed similar PROMs when recording the Lower Extremity Activity Scale [33] and Forgotten Joint Score‐12 [31] in both the cemented and uncemented groups. These PROMs could not be directly compared due to the different scoring systems used. Patient demographics were included in each of the studies but only Boyle et al. [4] recorded co‐morbidities such as smoking and diabetes. Other factors such as operating time and prophylactic antibiotics were not taken into account in any of the studies.

In conclusion, this meta‐analysis demonstrated a significant decrease in all‐cause revisions and revisions for aseptic loosening when comparing uncemented to the cemented primary TKA. A statistically significant difference was shown in the two studies [2, 36] including morbidly obese patients but was not shown in Boyle et al. [4] which included patients with a mean BMI of 37.4. However, due to the limited number of studies included, there is a likelihood that the true effect may differ significantly from the estimates shown in Figures 2 and 3. We have shown that the use of cementless TKA in obese patients is beneficial; however, future RCTs adjusted for confounding variables would be of benefit to corroborate these findings.

## AUTHOR CONTRIBUTIONS

Bernard Hendrick van Duren conceived the study. Bernard Hendrick van Duren and Giang Truong Le were responsible for the study design and analysis. Giang Troung Le was responsible for data collection. All authors have made substantial revisions to earlier drafts and approved the final manuscript.

## CONFLICT OF INTEREST STATEMENT

The authors declare no conflict of interest.

## ETHICS STATEMENT

Ethics approval was not required as a literature‐based study.

## Data Availability

Available as per literature search.
